# Characterization of left ventricular myocardial sodium-glucose cotransporter 1 expression in patients with end-stage heart failure

**DOI:** 10.1186/s12933-020-01141-1

**Published:** 2020-09-30

**Authors:** Alex Ali Sayour, Attila Oláh, Mihály Ruppert, Bálint András Barta, Eszter Mária Horváth, Kálmán Benke, Miklós Pólos, István Hartyánszky, Béla Merkely, Tamás Radovits

**Affiliations:** 1 Experimental Research Laboratory, Heart and Vascular Center, Semmelweis University, Városmajor u. 68., 1122 Budapest, Hungary; 2 Department of Physiology, Semmelweis University, Budapest, Hungary

**Keywords:** Sodium-glucose cotransporter 1, SGLT2 inhibitor, Dual SGLT1/2 inhibitors, Heart failure, Cardiac resynchronization therapy

## Abstract

**Background:**

Whereas selective sodium-glucose cotransporter 2 (SGLT2) inhibitors consistently showed cardiovascular protective effects in large outcome trials independent of the presence of type 2 diabetes mellitus (T2DM), the cardiovascular effects of dual SGLT1/2 inhibitors remain to be elucidated. Despite its clinical relevance, data are scarce regarding left ventricular (LV) SGLT1 expression in distinct heart failure (HF) pathologies. We aimed to characterize LV SGLT1 expression in human patients with end-stage HF, in context of the other two major glucose transporters: GLUT1 and GLUT4.

**Methods:**

Control LV samples (Control, n = 9) were harvested from patients with preserved LV systolic function who went through mitral valve replacement. LV samples from HF patients undergoing heart transplantation (n = 71) were obtained according to the following etiological subgroups: hypertrophic cardiomyopathy (HCM, n = 7); idiopathic dilated cardiomyopathy (DCM, n = 12); ischemic heart disease without T2DM (IHD, n = 14), IHD with T2DM (IHD + T2DM, n = 11); and HF patients with cardiac resynchronization therapy (DCM:CRT, n = 9, IHD:CRT, n = 9 and IHD-T2DM:CRT, n = 9). We measured LV SGLT1, GLUT1 and GLUT4 gene expressions with qRT-PCR. The protein expression of SGLT1, and activating phosphorylation of AMP-activated protein kinase (AMPKα) and extracellular signal-regulated kinase 1/2 (ERK1/2) were quantified by western blotting. Immunohistochemical staining of SGLT1 was performed.

**Results:**

Compared with controls, LV SGLT1 mRNA and protein expressions were significantly and comparably upregulated in HF patients with DCM, IHD and IHD + T2DM (all P < 0.05), but not in HCM. LV SGLT1 mRNA and protein expressions positively correlated with LVEDD and negatively correlated with EF (all P < 0.01). Whereas AMPKα phosphorylation was positively associated with SGLT1 protein expression, ERK1/2 phosphorylation showed a negative correlation (both P < 0.01). Immunohistochemical staining revealed that SGLT1 expression was predominantly confined to cardiomyocytes, and not fibrotic tissue. Overall, CRT was associated with reduction of LV SGLT1 expression, especially in patients with DCM.

**Conclusions:**

Myocardial LV SGLT1 is upregulated in patients with HF (except in those with HCM), correlates significantly with parameters of cardiac remodeling (LVEDD) and systolic function (EF), and is downregulated in DCM patients with CRT. The possible role of SGLT1 in LV remodeling needs to be elucidated.

## Introduction

Selective sodium-glucose cotransporter 2 (SGLT2) inhibitors are novel oral antidiabetic agents that block SGLT2 in the proximal convoluted tubule of the kidney, resulting in increased glucose excretion. Recent cardiovascular outcome trials in patients with type 2 diabetes mellitus (T2DM) [1–3], and dedicated heart failure (HF) trials in patients with HF and reduced ejection fraction (HFrEF) with or without T2DM [4–6] have demonstrated a consistently significant reduction in hospitalization for HF with SGLT2 inhibitor treatment versus placebo. Therefore, it has been postulated that SGLT2 inhibition in the kidney does not serve as full explanation for the marked clinical benefits associated with SGLT2 inhibitor treatment [7–11], suggesting direct cardiovascular mechanisms, which are currently incompletely understood given that SGLT2 is not expressed in the normal or diseased heart [9, 12–17].

In contrast, both healthy and diseased hearts express high levels of SGLT1 [17–19], which has been identified as a major glucose transporter in the myocardium besides the facilitative glucose transporters 1 and 4 (GLUT1 and 4) in humans and rodents [17–23]. The myocardial expression of SGLT1 in humans is altered in various cardiovascular disease states [16, 18, 24]. The currently marketed SGLT2 inhibitors are selective for SGLT2 over SGLT1 to different extent, of which canagliflozin is capable of slightly inhibiting SGLT1 in the small intestine at clinically relevant doses [25, 26]. In fact, dual SGLT1/2 inhibitors have been developed to reduce postprandial glucose absorption via blockade of SGLT1 in the small intestine, while at the same time preserving the beneficial renal glucosuric effect by SGLT2 inhibition [27]. Individuals carrying loss-of-function mutations in the SGLT1 gene are estimated to have lower risk for developing HF, driven by mitigation of postprandial hyperglycemic episodes [28]. The dual SGLT1/2 inhibitor sotagliflozin is currently being tested in cardiovascular outcome trial in patients with T2DM [27, 29].

Despite its substantial clinical relevance, there is a gap of knowledge regarding the physiological and pathophysiological roles of SGLT1 in the myocardium [30]. Some studies in small animals suggested that pharmacological SGLT1/2 inhibition could exacerbate myocardial dysfunction following ischemia–reperfusion injury by inhibiting glucose uptake [19, 21]. In cases when SGLT1 is upregulated in the heart, dual SGLT1/2 inhibitors or even SGLT2 inhibitors might affect myocardial SGLT1, but this is currently unclear [8]. Therefore, its expression needs to be further characterized in patients with end-stage HF of distinct etiologies in order to better predict and understand the putative direct cardiac effects of SGLT1 inhibition in these patient groups. Accordingly, we aimed to quantify LV SGLT1 expression in context of GLUT1 and GLUT4 in a relatively large number of samples from human patients with end-stage HF.

## Materials and methods

Well-characterized, de-identified human myocardial tissue samples were obtained from the Transplantation Biobank of the Heart and Vascular Center at Semmelweis University, Budapest, Hungary [31, 32]. The procedure of sample procurement was reviewed and approved by the institutional and national ethics committee (ethical permission numbers: ETT TUKEB 7891/2012/EKU (119/PI/12.) and TUKEB 73/2005.). Informed consent was obtained from patients in line with the Declaration of Helsinki prior to sample collection. In all cases, myocardial LV samples were surgically removed and immediately snap-frozen in liquid nitrogen under sterile conditions for molecular measurements, whereas LV samples for histological analyses were immediately conserved in 4% buffered paraformaldehyde. Control myocardial LV samples (n = 9) were isolated from LV papillary muscles removed from patients undergoing mitral valve replacement (open procedure) due to mitral regurgitation. Myocardial LV samples from end-stage HF patients (n = 71) were collected during heart transplantation (HTx) from the diseased hearts of the recipients immediately after explantation. Echocardiography data registered prior to surgery were obtained from the database of our Transplantation Biobank.

### Study population

An overall of 80 LV samples were included in the present study. End-stage HF patients were stratified into subgroups based on the etiology of HF, and whether cardiac resynchronization therapy (CRT) had been received up to the time of HTx. Accordingly, the following groups were defined in our study who met the outlined criteria:

1. Controls (n = 9): preserved LV systolic function; absence of myocardial disease
2. End-stage HF patients not receiving CRT (n = 44):
  - Hypertrophic cardiomyopathy (HCM, n = 7): severe LV hypertrophy; absence of relevant comorbidities (hypertension, T2DM); no relevant coronary atherosclerosis
  - Dilated cardiomyopathy (DCM, n = 12): severe LV dilation not explained by valvular disease; no history of myocarditis; no relevant comorbidities (hypertension, T2DM); no relevant coronary atherosclerosis
  - Ischemic heart disease (IHD, n = 14): severe diffuse coronary atherosclerosis at multiple sites, with or without prior revascularization therapy; no T2DM as comorbidity
  - Ischemic heart disease and T2DM (IHD-T2DM, n = 11): severe diffuse coronary atherosclerosis at multiple sites, with or without prior revascularization therapy; T2DM as comorbidity
3. End-stage HF patients receiving CRT (n = 27):
  - DCM with CRT (CRT:DCM, n = 9): severe LV dilation not explained by valvular disease; no history of myocarditis; no relevant comorbidities (hypertension, T2DM); no relevant coronary atherosclerosis
  - IHD with CRT (CRT:IHD, n = 9): severe diffuse coronary atherosclerosis at multiple sites, with or without prior revascularization therapy; no T2DM as comorbidity
  - IHD and T2DM, with CRT (CRT:IHD-T2DM, n = 9): severe diffuse coronary atherosclerosis at multiple sites, with or without prior revascularization therapy; T2DM as comorbidity

Several aspects—besides HF etiology—were considered when selecting HF patients. First, because left ventricular assist device (LVAD) therapy has positive effects on LV molecular signaling [33], we took care not to include patients who were on LVAD prior to HTx. Second, it is important to separate end-stage HF patients within the same etiology based on whether or not CRT was received, because CRT beneficially affects LV morphology and function, as well as LV molecular signaling in patients with HF [34]. However, none of the HCM patients received CRT. Third, we could identify T2DM patients only with IHD, possibly because at this advanced stage of HF, definitive T2DM might have been associated with coronary lesions (i.e. could not be classified as diabetic cardiomyopathy).

Prior to cardiac surgery, echocardiographic measurements were performed. Left ventricular end-diastolic diameter (LVEDD, mm) was quantified as a marker of LV dilation, a hallmark of LV adverse remodeling. Systolic function was determined by LV ejection fraction (EF, %) based on the Simpson method. LV fractional shortening (FS, %) was calculated from LVEDD and LV end-systolic diameter (LVESD) as: [(LVEDD − LVESD)/LVEDD]*100.

### RNA isolation and quality control

Myocardial LV tissue samples (~ 25 mg) were homogenized in Buffer RLT (Qiagen, Netherlands) using Bertin Precellys 24 Tissue Homogenizer with Bertin Cryolys cooling system (Bertin Technologies, France) to ensure adequate and constant cooling (~ 0 °C) of samples throughout the procedure. Then, total RNA was isolated using RNeasy Fibrous Tissue Kit (Qiagen) as per the manufacturer’s protocol. RNA concentration was measured photometrically at 260 nm, while RNA purity was ensured by obtaining 260/280 nm and 260/230 nm optical density ratio of ~ 2.0, respectively. Each individual RNA sample was loaded onto Agilent 6000 Pico LabChips (Agilent Technologies, Santa Clara, CA, USA) and analysed using Agilent 2100 Bioanalyzer. Based on the ratio of 18S/28S rRNA in the electrophoretogram of each sample, an RNA Integrity Number (RIN) was assigned (ranging from RIN 0–10, higher RIN values indicate excellent RNA quality). The RIN of samples homogenized from intraoperatively obtained human tissues typically range from ~ 6.0–8.0 when efforts are made to prevent degradation [35]. In the present study all study groups had a mean RIN > 7.9, indicating excellent RNA quality of samples which ensures that RNA degradation does not explain the differences in mRNA expressions among the samples.

### Quantitative real-time polymerase chain reaction

Reverse transcription of RNA to cDNA was conducted with QuantiTect Reverse Transcription Kit (Qiagen) by using 1 μg RNA of each sample and random primers, as per protocol. Then, quantitative real-time polymerase chain reaction (qRT-PCR) was performed on StepOnePlus RT PCR System (Thermo Fisher Scientific, Waltham, MA, USA) using TaqMan Universal PCR MasterMix and TaqMan Gene Expression Assays (Thermo Fisher Scientific) for the following targets: Solute Carrier Family 5 Member 1 (SLC5A1 encoding SGLT1; ID: Hs01573793_m1); SLC5A2 (encoding SGLT2; ID: Hs00894642_m1); SLC2A1 (encoding GLUT1; ID: Hs00892681_m1); SLC2A4 (encoding GLUT4; ID: Hs00168966_m1); and glyceraldehyde-3-phosphate dehydrogenase (GAPDH; ID: Hs99999905_m1). Every sample was quantified in duplicates or triplicates in a volume of 10 μl in each well containing 1 μl cDNA. Data were normalized to the housekeeping GAPDH, then to a positive calibrator (a pool of cDNA from all samples of the DCM group) in each case. Accordingly, gene expression levels were calculated using the comparative method (2^−ΔΔCT^).

### Western blot analysis

Myocardial LV tissue samples were homogenized in RIPA buffer (Bio-Rad Laboratories, Hercules, CA, USA) containing protease and phosphatase inhibitor cocktail (Roche, Basel, Switzerland), using Bertin Precellys 24 Tissue Homogenizer with Bertin Cryolys cooling system (Bertin Technologies). The concentrations of the extracted proteins were measured by BCA assay (Thermo Fisher Scientific). Then, protein homogenates were suspended in sample buffer and heated at 70 °C for 10 min. A total of 40 µg protein for each sample was loaded onto 6–12% acrylamide gels and separated with sodium dodecyl sulphate polyacrylamide gel electrophoresis system (Bio-Rad Laboratories). Gels were transferred to polyvinylidene fluoride membranes under dry conditions. Membranes were then washed and blocked for 1 h in 5% bovine serum albumin (BSA) in Tris-buffered saline Tween 20 (TBST) at room temperature. Next, membranes were incubated overnight at 4 °C with the following primary antibodies diluted in 2.5% BSA in TBST (purchased from Cell Signaling Technology, Danvers, MA, USA): SGLT1 (1:1000; ID: #5042); phosphorylated adenosine-monophosphate-activated protein kinase α catalytic subunit (P-AMPKα, Thr172) (1:1000; ID: #2535); total-AMPKα (1:1000; ID: #2532); phosphorylated extracellular signal-regulated protein kinase 1/2 (P-ERK1/2, Thr202/Tyr204) (1:1000; ID: #9101); total-ERK1/2 (1:1000; ID: #9102) and the housekeeping GAPDH (1:5000; ID: #5174). The blots were washed and incubated with horseradish peroxidase-conjugated secondary antibody (1:5000, 2.5% BSA in TBST) for 1 h at room temperature. The immunoreactive protein bands were developed using Super Signal West Pico Plus (Thermo Fisher Scientific) chemiluminescent substrate. The intensity of the immunoblot bands was analyzed with Bio-Rad Image Lab Software 6.0 (Bio-Rad Laboratories). The intensity of the bands of the primary targets was normalized to that of the housekeeping GAPDH on the same blot.

### Immunohistochemistry

Immunohistochemistry was performed as previously described [36]. Briefly, following fixation in 4% buffered paraformaldehyde for ~ 24 h, LV samples were embedded in paraffin and 7 μm thick sections were cut. After deparaffination and antigen retrieval, sections were incubated with anti-SGLT1 antibody (1:100; overnight, 4 °C; ab14686). HRP-conjugated secondary antibody (30 min, room temperature) and black colored nickel–cobalt enhanced diaminobenzidine (6 min, room temperature) were used to visualize the labeling. Light microscopic examination was performed using Nikon Eclipse Ni Microscope (Nikon Instruments, Amstelveen, Netherlands) and a digital image was captured in each section (from each patient) using Nikon DS-RI2 camera (Nikon Instruments) with 40 × dry objective.

Immunofluorescent staining was performed after deparaffination and antigen retrieval using anti-SGLT1 antibody (1:100; overnight at 4 °C; ab14686). Alexa-Fluor 488 conjugated goat anti-rabbit IgG (1:500; 30 min, room temperature; ab150077) served as secondary antibody. Sodium–potassium ATP-ase (Na–K-ATPase) was labeled by anti-alpha 1 Sodium Potassium ATPase antibody (1:200, 2 h, room temperature; ab7671), where Alexa-Fluor 568 goat anti-mouse IgG (1:500; 30 min, room temperature; ab175473) was used for visualization. Then, slides were covered by 4′,6-diamidino-2-phenylindole (DAPI)-containing mounting medium (Vectashield; Vector Laboratories, Burlingame, CA, USA). Representative images were acquired by Nikon Eclipse A1 Confocal Laser Microscope (Nikon Instruments) using a 40× dry objective.

All antibodies used for immunohistochemical measurements were purchased from Abcam, Cambridge, UK.

### Statistical analysis

Values are expressed as mean ± SEM for continuous variables, whereas categorical variables are expressed as frequencies and percentages. The assumption of normal distribution of the data was analyzed using the Shapiro–Wilk test and the predicted probability (P-P) plots, and when violated, log2-transformed data were used to analyze group differences. The assumptions of normal distribution and homoscedasticity of the residuals were analyzed by plotting the predicted values and residuals on scatter plots. Significance of difference between two groups was assessed using unpaired Student *t*-test with Welch’s correction. To compare means of five groups, one-way analysis of variance (ANOVA) was performed with Welch’s correction followed by Dunnett T3 post hoc test to compute intergroup differences relative to the Control group.

Analysis of covariance (ANCOVA) was performed to quantify the observed differences after adjusting for age, sex, and body mass index (BMI). The assumption of homogeneity of regression slopes was not violated in any case as indicated by non-significant interaction between the covariates and the fixed factor. Also, substantial collinearity among the predictor variables was not an issue as variance inflation factors (VIF) were < 5.00 in all cases. Reported P values associated with bias-corrected and accelerated (BCa) 95% confidence intervals (CI) based on n = 1000 bootstrap samples were adjusted for multiple comparisons using Bonferroni correction.

For zero-order correlation analysis, Spearman’s *rho* (r_s_) was computed. We estimated that at two-tailed α = 0.05 and power (β) of 0.8, in order to detect a medium effect size with partial correlation analysis based on 4 predictors, a sample size of n = 55 is required. Partial correlation analysis was performed on ranked scores to compute correlation coefficients adjusted for age, sex, and BMI. For all correlation coefficients, BCa 95% CI are reported based on n = 1000 bootstrap samples.

Point-biserial correlation analysis on ranked scores was performed to compute the overall effect of CRT on LV mRNA expression of target genes.

Statistical analyses were carried out using IBM SPSS Statistics 25 (IBM, Armonk, NY, USA) and GraphPad Prism 8 (GraphPad Software, San Diego, CA, USA), the latter was also used to graph data. In all cases, the untransformed, original datapoints are graphed. A two-tailed P < 0.05 value was considered statistically significant.

## Results

### Study populations

Patient characteristics in each group are provided in Table 1. As seen, controls had preserved LV systolic function (EF = 61.2 ± 3.4%), while the HF groups, including those with HCM, presented with severely reduced EFs.

**Table 1:** Patient baseline characteristics and RNA Integrity Numbers (RIN) of myocardial left ventricular RNA samples according to subgroups

	Control (n = 9)	HCM (n = 7)	DCM (n = 12)	IHD (n = 14)	IHD-T2DM (n = 11)	CRT:DCM (n = 9)	CRT:IHD (n = 9)	CRT:IHD-T2DM (n = 9)
Age (years)	68.6 ± 1.9	36.6 ± 4.4	46.8 ± 3.4	58.7 ± 1.4	57.0 ± 1.4	47.7 ± 4.1	59.0 ± 1.6	60.1 ± 1.6
Sex (F, %)	8/9 (89%)	4/7 (57%)	2/12 (17%)	5/12 (36%)	3/11 (27%)	3/9 (33%)	0/9 (0%)	2/9 (22%)
BMI (kg/m^2^)	26.1 ± 1.6	25.5 ± 1.9	25.7 ± 1.6	26.5 ± 0.8	27.9 ± 0.9	23.5 ± 1.0	27.7 ± 1.6	30.0 ± 1.1
LVEDD (mm)	53.2 ± 4.0	50.7 ± 4.6	73.4 ± 2.6	69.9 ± 2.7	63.9 ± 2.7	76.2 ± 3.8	70.6 ± 6.2	70.0 ± 3.6
EF (%)	61.2 ± 3.4	36.9 ± 4.6	21.9 ± 1.1	27.3 ± 1.4	22.9 ± 1.9	19.3 ± 2.7	19.7 ± 2.4	23.3 ± 2.4
FS (%)	32.1 ± 3.8	20.7 ± 3.5	10.9 ± 0.9	14.6 ± 1.6	13.0 ± 2.5	8.2 ± 2.5	14.7 ± 3.7	15.5 ± 1.4
Years with CRT	–	–	–	–	–	2.9 ± 0.6	3.2 ± 1.8	3.4 ± 0.8
BB	8/9 (89%)	5/7 (71%)	9/12 (75%)	11/14 (79%)	4/11 (36%)	2/9 (22%)	8/9 (89%)	8/9 (89%)
ACEi or ARB	1/9 (11%)	0/7 (0%)	7/12 (58%)	11/14 (79%)	9/11 (82%)	3/9 (33%)	7/9 (78%)	8/9 (89%)
MRA	2/9 (22%)	4/7 (57%)	9/12 (75%)	13/14 (89%)	8/11 (73%)	6/9 (67%)	8/9 (89%)	6/9 (67%)
Diuretic	4/9 (44%)	4/7 (57%)	10/12 (83%)	11/14 (79%)	7/11 (64%)	7/9 (78%)	7/9 (78%)	6/9 (67%)
Statin	3/9 (33%)	0/7 (0%)	2/12 (17%)	7/14 (50%)	6/11 (55%)	0/9 (0%)	4/9 (44%)	8/9 (89%)
SU	–	–	–	–	2/11 (18%)	–	–	2/9 (22%)
Metformin	–	–	–	–	6/11 (55%)	–	–	5/9 (55%)
DPP4i	–	–	–	–	4/11 (36%)	–	–	1/9 (11%)
Insulin	–	–	–	–	3/11 (27%)	–	–	1/9 (11%)
RIN	9.3 ± 0.1	8.3 ± 0.4	8.4 ± 0.3	8.1 ± 0.4	8.2 ± 0.3	7.9 ± 0.3	8.0 ± 0.3	7.9 ± 0.3

*HCM* hypertrophic cardiomyopathy, *DCM* dilated cardiomyopathy, *IHD* ischemic heart disease, *T2DM* type 2 diabetes mellitus, *CRT* cardiac resynchronization therapy, *BMI* body mass index, *LVEDD* left ventricular end-diastolic diameter, *EF* left ventricular ejection fraction, *FS* fractional shortening, *BB* beta blocker, *ACEi* angiotensin converting enzyme inhibitor, *ARB* angiotensin II receptor blocker, *MRA* mineralocorticoid receptor antagonist, *SU* sulfonylurea, *DPP4i* dipeptidyl peptidase-4 inhibitor, *RIN* RNA integrity number

### Left ventricular mRNA expression profiles of SGLT1, SGLT2, GLUT1 and GLUT4

**Fig. 1:**
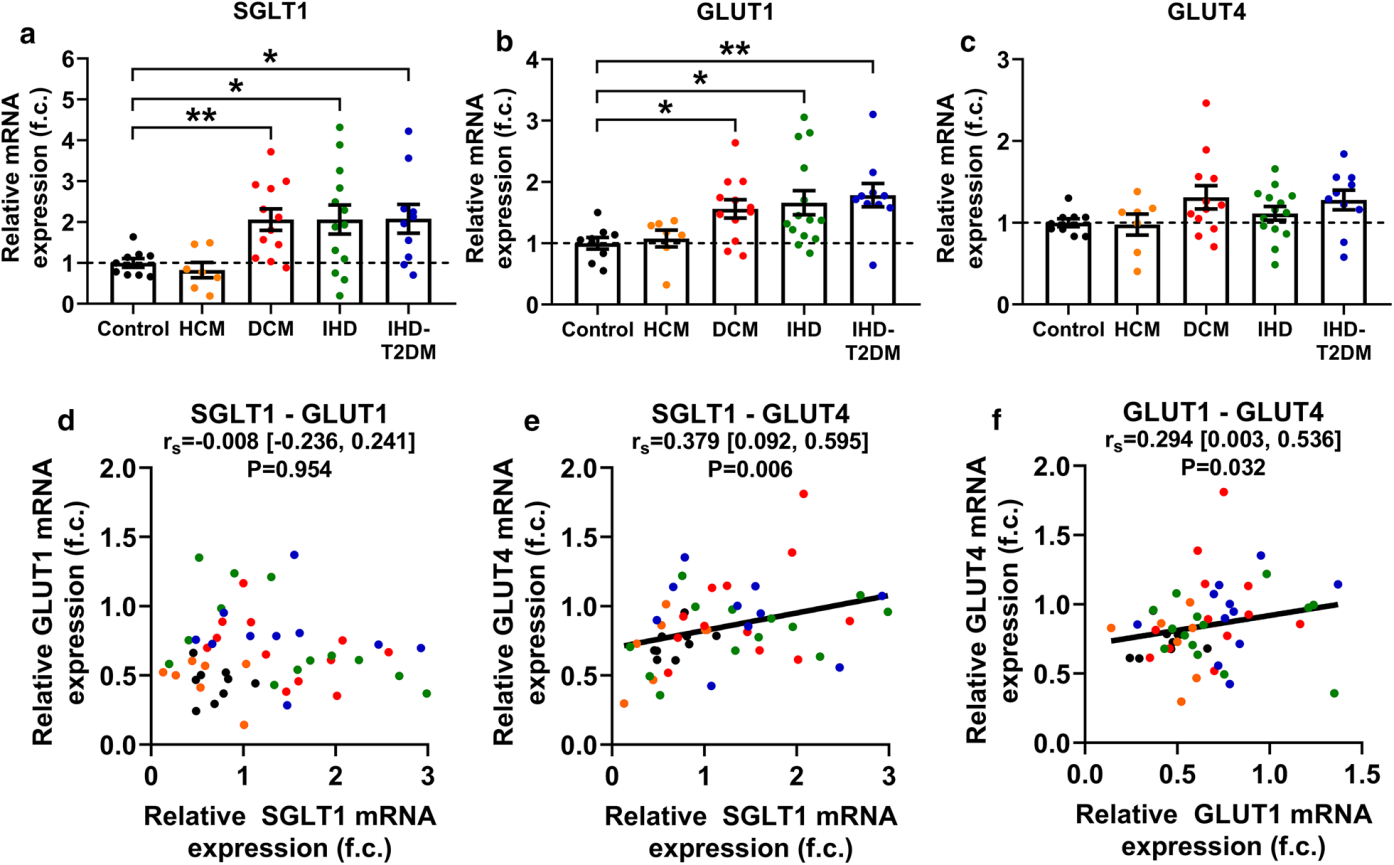
Myocardial left ventricular expression of the three major glucose transporters and their correlations. **a** Quantification of left ventricular (LV) relative mRNA expression of sodium-glucose cotransporter 1 (SGLT1) in controls and in patients with heart failure (HF). **b** Quantification of LV relative mRNA expression of facilitative glucose transporter 1 (GLUT1) in controls and in patients with HF. **c** Quantification of LV relative mRNA expression of GLUT4 in controls and in patients with HF. **d** Correlation between the LV relative mRNA expressions of SGLT1 and GLUT1. **e** Correlation between the LV relative mRNA expressions of SGLT1 and GLUT4. **f** Correlation between the LV relative mRNA expressions of GLUT1 and GLUT4. For easier interpretation, expression values are normalized to that of Controls (i.e. mean of Control group = 1.00). To compare means of five groups, one-way analysis of variance (ANOVA) was performed with Welch’s correction followed by Dunnett T3 post hoc test to compute intergroup differences relative to the Control group. Statistical significance according to the post hoc test (relative to Control) is highlighted as follows: *P < 0.05, **P < 0.01. For zero-order correlation analysis, Spearman’s *rho* (r_s_) was computed. For all correlation coefficients, bias-corrected and accelerated (BCa) 95% confidence intervals (CI) are reported based on n = 1000 bootstrap samples. *DCM* dilated cardiomyopathy, *f. c.* fold change, *GLUT1 and 4* facilitative glucose transporter 1 and 4, *HCM* hypertrophic cardiomyopathy, *IHD* ischemic heart disease, *IHD-T2DM* IHD and type 2 diabetes mellitus, *r*_*s*_ Spearman’s *rho* (correlation coefficient), *SGLT1* sodium-glucose cotransporter 1

Myocardial LV mRNA expression of SGLT1 significantly differed among groups based on cardiac pathology (ANOVA P = 0.004) (Fig. 1a). Compared with controls, pairwise comparisons revealed that SGLT1 was significantly upregulated in patients with DCM (P = 0.007) but not with HCM (P = 0.831) (Fig. 1a). Those with IHD also had a significantly increased SGLT1 expression irrespective of T2DM (P < 0.05, respectively) (Fig. 1a). According to ANCOVA, differences in LV SGLT1 expression persisted even after adjusting for age, sex, and BMI (P = 0.024) (Table 2, part A). Also, based on estimated marginal means and bootstrapped, Bonferroni-corrected P values, DCM (P = 0.020) and IHD (P = 0.040) groups had a significantly increased SGLT1 expression compared to controls, while there was a strong tendency in case of IHD-T2DM (P = 0.056) (Table 2, part A).

**Table 2:** Results of statistical analyses correcting for age, sex, and body mass index (BMI)

A: Analysis of covariance (correcting for age, sex, and body mass index)							
	Model summary		Control	HCM	DCM	IHD	IHD-T2DM
SGLT1 expression	P = 0.024	EM mean (± SEM)	0.355 ± 0.267	0.926 ± 0.339	1.654 ± 0.234	1.332 ± 0.235	1.391 ± 0.258
		P value (vs. Control)	–	0.920	0.020	0.040	0.056
GLUT1 expression	P = 0.035	EM mean (± SEM)	0.526 ± 0.091	0.402 ± 0.120	0.630 ± 0.081	0.754 ± 0.089	0.801 ± 0.077
		P value (vs. Control)	–	1.000	1.000	0.224	0.116
GLUT4 expression	P = 0.544	EM mean (± SEM)	0.755 ± 0.108	0.818 ± 0.114	0.950 ± 0.105	0.789 ± 0.071	0.896 ± 0.073
		P value (vs. Control)	–	1.000	0.840	1.000	1.000

B: Partial correlation analysis (correcting for age, sex, and body mass index)		
	Partial correlation coefficient (BCa 95% CI)	P value
LVEDD–SGLT1	0.476 [0.182, 0.700]	0.002
EF–SGLT1	− 0.542 [− 0.755, − 0.233]	< 0.001
LVEDD–GLUT1	− 0.036 [− 0.365, 0.301]	0.819
EF–GLUT1	− 0.147 [− 0.455, 0.165]	0.331
LVEDD–GLUT4	0.204 [− 0.138, 0.503]	0.195
EF–GLUT4	− 0.141 [− 0.411, 0.134]	0.350
SGLT1–GLUT1	− 0.060 [− 0.311, 0.226]	0.693
SGLT1–GLUT4	0.324 [− 0.011, 0.604]	0.030
GLUT1–GLUT4	0.254 [0.004, 0.503]	0.085

A: Results of analysis of covariance (ANCOVA): left ventricular (LV) relative mRNA expressions of the three major glucose transporters are corrected for age, sex, and BMI according to study subgroups. The related P values are reported as model summary. Estimated marginal (EM) means are provided. To test intergroup differences, we report P values associated with bias-corrected and accelerated (BCa) 95% confidence intervals (CI) based on n = 1000 bootstrap samples, that are adjusted for multiple comparisons using Bonferroni correction

B: Results of partial correlation analysis on ranked scores: partial correlation coefficients (r) of LV relative mRNA expression of the three major glucose transporters and echocardiographic parameters are corrected for age, sex, and BMI. For all partial correlation coefficients, bias-corrected and accelerated (BCa) 95% confidence intervals (CI) are reported based on n = 1000 bootstrap samples

*HCM* hypertrophic cardiomyopathy, *DCM* dilated cardiomyopathy, *IHD* ischemic heart disease, *T2DM* type 2 diabetes mellitus, *SGLT1* sodium-glucose cotransporter-1, *GLUT1/4* facilitated glucose transporter 1/4, *EM mean* estimated marginal mean, *LVEDD* left ventricular end-diastolic diameter, *EF* left ventricular ejection fraction, *BCa 95% CI* bias-corrected and accelerated 95% confidence interval

We found no detectable LV SGLT2 mRNA expression in any of the studied groups (data not depicted).

LV mRNA expression of GLUT1 also differed significantly among the studied groups (ANOVA P = 0.011) (Fig. 1b). Patients with DCM, IHD and IHD-T2DM had a significantly increased GLUT1 expression as compared with controls (P < 0.05, respectively), but not those with HCM (P = 1.000) (Fig. 1b). ANCOVA revealed that GLUT1 was still significantly different among the groups after adjusting for age, sex, and BMI (P = 0.035) (Table 2, part A). However, intergroup differences were not statistically significant (Table 2, part A).

Finally, GLUT4 expression was comparable among the groups (ANOVA P = 0.131) (Fig. 1c), even after adjusting for age, sex, and BMI (ANCOVA P = 0.544) (Table 2, part A).

### Correlation of LV mRNA expressions of the three major myocardial glucose transporters

Despite being similarly upregulated in DCM, IHD and IHD-T2DM patients compared to controls, LV mRNA expression of SGLT1 did not significantly correlate with that of GLUT1 (r_s_ = -0.008, P = 0.954) (Fig. 1d). However, GLUT4 mRNA expression showed positive significant correlation with SGLT1 (r_s_ = 0.379, P = 0.006) (Fig. 1e) and GLUT1 (r_s_ = 0.294, P = 0.032) (Fig. 1f), respectively.

After adjusting for age, sex, and BMI, GLUT4 mRNA expression remained significantly correlated with that of SGLT1 (r = 0.324, P = 0.030), and also tended to correlate with that of GLUT1 (r = 0.254, P = 0.085) (Table 2, part B). The adjustment did not meaningfully affect the absence of correlation between SGLT1 and GLUT1 mRNA expressions (r = -0.060, P = 0.693) (Table 2, part B).

### Correlation of LV mRNA expressions with LVEDD, EF and FS

**Fig. 2:**
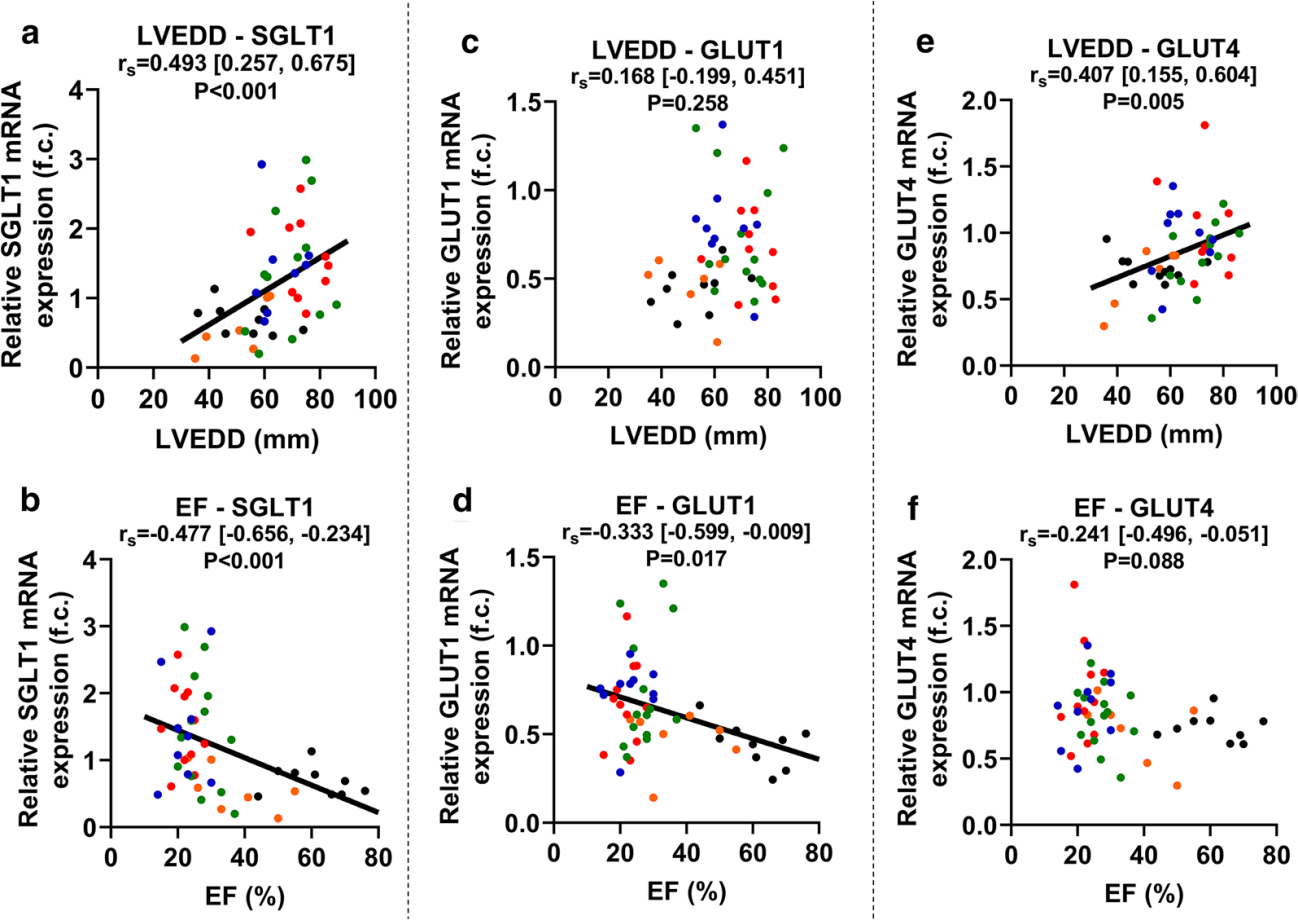
Correlation between glucose transporter mRNA expressions and echocardiographic parameters registered prior to sample procurement. **a** Correlation between left ventricular (LV) end-diastolic diameter (LVEDD) and LV sodium-glucose cotransporter 1 (SGLT1) relative mRNA expression. **b** Correlation between LV ejection fraction (EF) and LV SGLT1 relative mRNA expression. **c** Correlation between LVEDD and LV facilitative glucose transporter 1 (GLUT1) relative mRNA expression. **d** Correlation between LV EF and LV GLUT1 relative mRNA expression. **e** Correlation between LVEDD and LV GLUT4 relative mRNA expression. **f** Correlation between LV EF and LV GLUT4 relative mRNA expression. The color of dots corresponds to that of Fig. 1 according to the subgroups. For zero-order correlation analysis, Spearman’s *rho* (r_s_) was computed. For all correlation coefficients, bias-corrected and accelerated (BCa) 95% confidence intervals (CI) are reported based on n = 1000 bootstrap samples. *EF* ejection fraction, *f. c.* fold change, *GLUT1 and 4* facilitative glucose transporter 1 and 4, *LVEDD* left ventricular end-diastolic diameter, *r*_*s*_ Spearman’s *rho* (correlation coefficient), *SGLT1* sodium-glucose cotransporter 1

LV SGLT1 mRNA expression showed a significant positive correlation with LVEDD (r_s_ = 0.493, P < 0.001), a marker of LV dilation (Fig. 2a). Furthermore, SGLT1 expression correlated negatively with parameters of LV systolic function: EF (r_s_ = -0.477, P < 0.001) (Fig. 2b) and FS (r_s_ = -0.542 [95% BCa CI -0.713, -0.272], P < 0.001), respectively. Partial correlation analysis on ranked scores revealed that after adjusting for age, sex, and BMI, SGLT1 mRNA expression remained significantly correlated with LVEDD (r = 0.476, P = 0.002), EF (r = − 0.542, P < 0.001) (Table 2, part B) and FS (r_s_ = − 0.644 [95% BCa CI − 0.775, − 0.533], P < 0.001), respectively.

LV GLUT1 mRNA expression showed significant inverse correlation with EF (r_s_ = − 0.333, P = 0.017) (Fig. 2d) and tended to correlate with FS (r_s_ = − 0.288 [95% BCa CI − 0.574, 0.066], P = 0.079), but not with LVEDD (Fig. 2c). On the contrary, GLUT4 expression correlated significantly with LVEDD (r_s_ = 0.407, P = 0.005) (Fig. 2e) and FS (r_s_ = − 0.449 [95% BCa CI − 0.713, − 0.118], P = 0.005), but only tendentially with EF (r_s_ = − 0.241, P = 0.088) (Fig. 2f). However, after adjusting for age, sex, and BMI, neither GLUT1 nor GLUT4 mRNA expression correlated significantly with LVEDD, EF (Table 2, part B) or FS, respectively.

### Protein expression of SGLT1 and phosphorylation of ERK1/2 and AMPKα

**Fig. 3:**
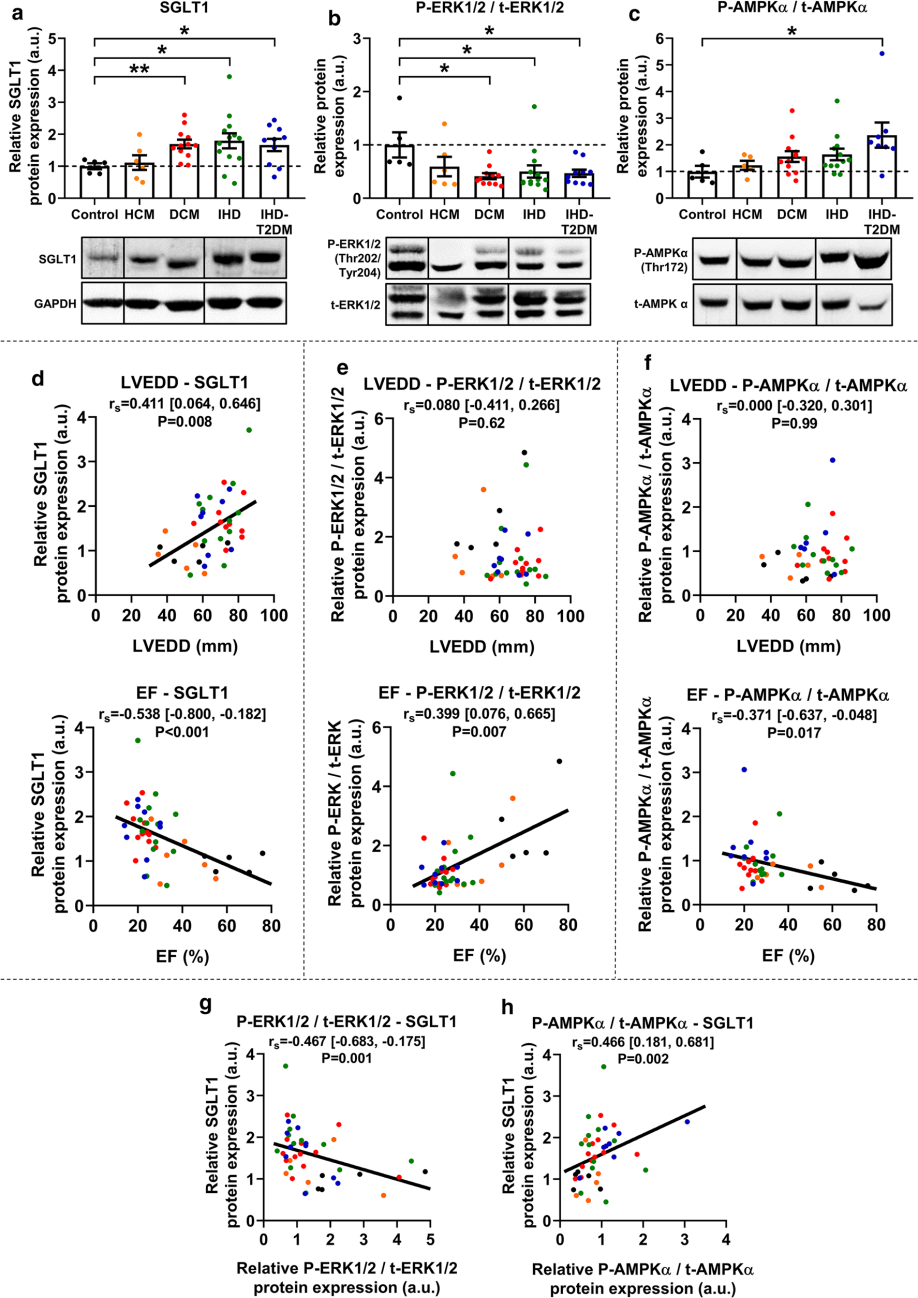
Protein expression of SGLT1, and activation of ERK1/2 and AMPKα. **a** Relative protein expression of left ventricular (LV) sodium-glucose cotransporter 1 (SGLT1) in controls and in patients with heart failure (HF). A representative blot is shown. **b** The ratio of LV phosphorylated extracellular signal-regulated kinase 1/2 (P-ERK1/2) versus total ERK1/2 (t-ERK1/2) in controls and in patients with HF. A representative blot is shown. **c** The ratio of LV phosphorylated adenosine-monophosphate-activated protein kinase α catalytic subunit (P-AMPKα) versus total AMPKα (t-AMPKα) in controls and in patients with HF. A representative blot is shown. **d** Correlation between LV end-diastolic diameter (LVEDD), ejection fraction (EF) and SGLT1 relative protein expression. **e** Correlation between LVEDD, EF and P-ERK1/2 / t-ERK1/2 relative protein expression. **f** Correlation between LVEDD, EF and P-AMPKα / t- AMPKα relative protein expression. **g** Correlation between P-ERK1/2 / t-ERK1/2 and SGLT1 relative protein expressions. **h** Correlation between P- AMPKα / t-AMPKα and SGLT1 relative protein expressions. For easier interpretation, expression values are normalized to that of Controls (i.e. mean of Control group = 1.00). To compare means of five groups, one-way analysis of variance (ANOVA) was performed with Welch’s correction followed by Dunnett T3 post hoc test to compute intergroup differences relative to the Control group. Statistical significance according to the post hoc test (relative to Control) is highlighted as follows: *P < 0.05, **P < 0.01. For zero-order correlation analysis, Spearman’s *rho* (r_s_) was computed. For all correlation coefficients, bias-corrected and accelerated (BCa) 95% confidence intervals (CI) are reported based on n = 1000 bootstrap samples. *AMPKα* adenosine-monophosphate-activated protein kinase α catalytic subunit, *DCM* dilated cardiomyopathy, *EF* ejection fraction, *ERK1/2* extracellular signal-regulated kinase 1/2, *f. c.* fold change, *HCM* hypertrophic cardiomyopathy, *LVEDD* left ventricular end-diastolic diameter, *IHD* ischemic heart disease, *IHD-T2DM* IHD and type 2 diabetes mellitus, *r*_*s*_ Spearman’s *rho* (correlation coefficient), *SGLT1* sodium-glucose cotransporter 1

Western blot analysis revealed that SGLT1 protein expression was significantly upregulated in patients with DCM, IHD, and IHD-T2DM (all P < 0.05) compared to controls, but not in those with HCM (Fig. 3a). LV SGLT1 protein expression showed a significant positive correlation with LVEDD (r_s_ = 0.411, P = 0.008) and a negative one with EF (r_s_ = − 0.583, P < 0.001) (Fig. 3d), similarly to mRNA expression.

The phosphorylation of ERK1/2 (also referred to as p44/42 mitogen-activated protein kinase, MAPK) on its activation sites (Thr202/Tyr204) was significantly downregulated in patients with DCM, IHD and IHD-T2DM (all P < 0.05) compared to controls, showing a reciprocal change in contrast to SGLT1 protein expression (Fig. 3b). While ERK1/2 phosphorylation was not associated with LVEDD (r_s_ = 0.080, P = 0.62), it showed significant positive correlation with EF (r_s_ = 0.399, P = 0.007) (Fig. 3e).

Compared to controls, the activating phosphorylation of AMPKα on Thr172 was numerically upregulated in patients with DCM (1.5-fold) and IHD (1.6-fold), without reaching statistical significance due to high variance (Fig. 3c). However, AMPKα phosphorylation was significantly increased in patients with IHD-T2DM (P = 0.036) (Fig. 3c). Similar to ERK1/2 phosphorylation, AMPKα phosphorylation was not related to LVEDD (r_s_ = 0.000, P = 0.99), however, negatively correlated with EF (r_s_ = − 0.371, P = 0.017) (Fig. 3f).

Phosphorylation of ERK1/2 negatively (P = 0.001), while phosphorylation of AMPKα positively (P = 0.002) correlated with SGLT1 protein expression (Fig. 3g, h).

### Histological assessment of myocardial SGLT1

**Fig. 4:**
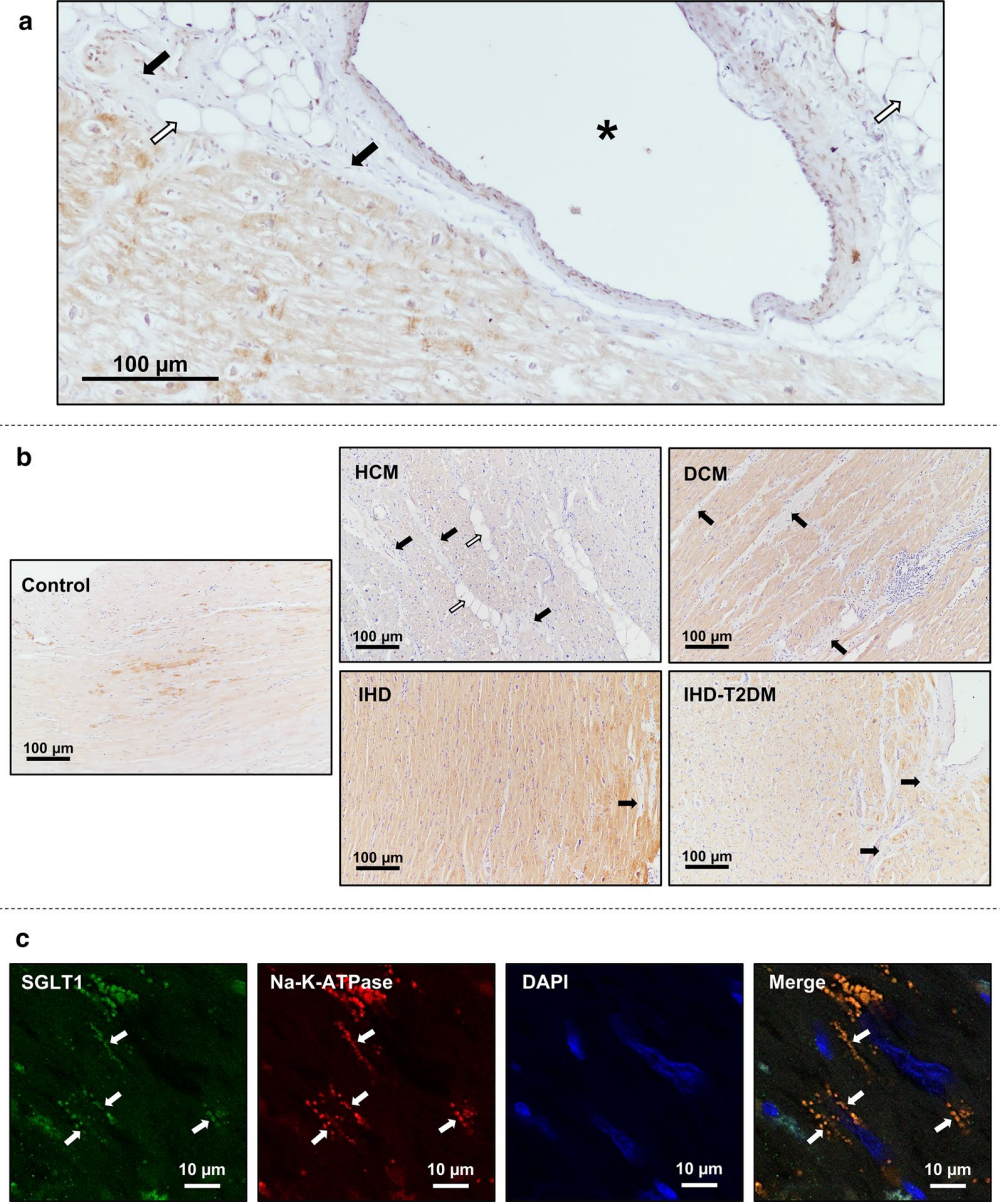
Immunohistochemical analysis of SGLT1 in the heart. **a** Representative left ventricular (LV) epicardial section from a patient with dilated cardiomyopathy (DCM). The brownish staining refers to sodium-glucose cotransporter 1 (SGLT1). Full arrows indicate fibrotic tissue, blank arrows indicate adipose tissue, whereas the asterisk marks the lumen of an epicardial vessel. **b** Representative LV sections from controls and from patients with heart failure (HF). The brownish staining refers to SGLT1. Full arrows indicate fibrotic tissue, whereas blank arrows indicate adipose tissue. **c** Representative LV immunofluorescent sections from a patient with DCM depicting SGLT1 in green, the sarcolemma marker sodium–potassium ATPase (Na–K-ATPase) in red, and nuclei (DAPI) in blue. A merge of these recordings is also shown. Full arrows indicate staining of SGLT1 and Na–K-ATPase, respectively. *DAPI* 4′,6-diamidino-2-phenylindole, *DCM* dilated cardiomyopathy, *HCM* hypertrophic cardiomyopathy, *IHD* ischemic heart disease, *IHD-T2DM* IHD and type 2 diabetes mellitus, *Na–K-ATPase* sodium–potassium ATPase, *SGLT1* sodium-glucose cotransporter 1

A representative LV epicardial histological section from a patient with DCM stained against SGLT1 is shown in Fig. 4a. The brownish staining of SGLT1 was predominantly confined to cardiomyocytes, whereas the staining of fibrotic and adipose tissues was negligible. A similar pattern was seen in the representative sections from patients in each study group (Fig. 4b).

Immunofluorescent staining of SGLT1 showed that its localization almost exclusively corresponded to that of Na–K-ATPase (Fig. 4c).

### Effect of CRT on the expression of SGLT1, GLUT1 and GLUT4

**Fig. 5:**
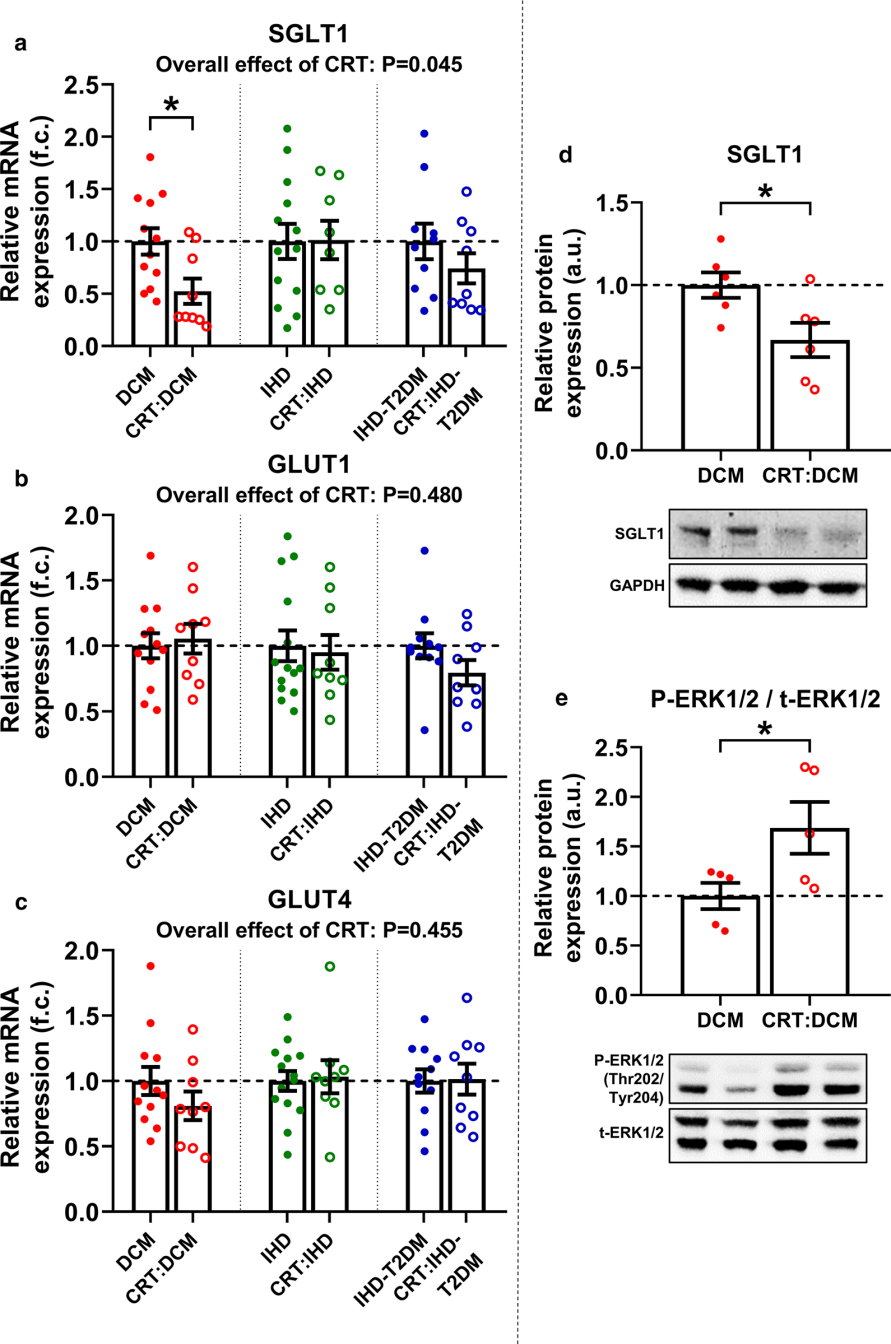
Effect of cardiac resynchronization therapy on left ventricular expression of the three major glucose transporters. **a** Comparison of left ventricular (LV) relative mRNA expression of sodium-glucose cotransporter 1 (SGLT1) between heart failure (HF) patients with and without cardiac resynchronization therapy (CRT). **b** Comparison of LV relative mRNA expression of facilitative glucose transporter 1 (GLUT1) between HF patients with and without CRT. **c** Comparison of LV relative mRNA expression of GLUT4 between HF patients with and without CRT. **d** Relative protein expression of LV SGLT1 in patients with dilated cardiomyopathy (DCM) with and without CRT. A representative blot is depicted. **e** Phosphorylated extracellular signal-regulated kinase 1/2 (P-ERK1/2) versus total ERK1/2 (t-ERK1/2) protein expressions in patients with DCM with and without CRT. A representative blot is depicted. For easier interpretation, expression values are normalized to that of non-CRT groups within each etiological subgroup (i.e. mean of corresponding non-CRT group = 1.00). Point-biserial correlation analysis on ranked scores was performed to compute the overall effect of CRT on LV mRNA expression of target genes, the related P values are reported. Significance of difference between two groups was assessed using unpaired Student *t*-test with Welch’s correction. Accordingly, statistical significance is highlighted as: *P < 0.05. *a.u.* arbitrary units, *CRT* cardiac resynchronization therapy, *DCM* dilated cardiomyopathy, *f. c.* fold change, *ERK1/2* extracellular signal-regulated kinase 1/2, *GAPDH* glyceraldehyde-3-phosphate dehydrogenase, *GLUT1 and 4* facilitative glucose transporter 1 and 4, *HCM* hypertrophic cardiomyopathy, *IHD* ischemic heart disease, *IHD-T2DM* IHD and type 2 diabetes mellitus, *SGLT1* sodium-glucose cotransporter 1

We investigated the effect of CRT on mRNA expression of SGLT1, GLUT1 and GLUT4 in LV samples from patients with DCM, IHD and IHD-T2DM. Overall, CRT was associated with significantly reduced LV SGLT1 expression (P = 0.045) (Fig. 5a). When comparing HF patients within the same etiological subgroup, we found CRT was associated with significantly decreased SGLT1 mRNA expression in DCM patients compared to those not receiving CRT (P = 0.026) (Fig. 5a). According to ANCOVA, this difference remained significant even after adjusting for age, sex, and BMI (estimated marginal means: 1.515 ± 0.205 vs. 0.774 ± 0.219, P = 0.048). SGLT1 mRNA expression was comparable among IHD patients with and without CRT, irrespective of T2DM (Fig. 5a), even after adjusting for age, sex, and BMI (estimated marginal means: IHD vs. CRT:IHD: 1.372 ± 0.241 vs. 1.563 ± 0.303, P = 0.642; IHD-T2DM vs. CRT:IHD-T2DM: 1.370 ± 0.287 vs. 1.147 ± 0.265, P = 0.576).

In line with mRNA expression, LV SGLT1 protein expression was significantly reduced in CRT:DCM patients, as compared with DCM patients not receiving CRT (P = 0.029) (Fig. 5d). The reciprocal upregulation of ERK1/2 phosphorylation was present in these patients (P = 0.045) (Fig. 5e).

CRT was not associated with significant differences in GLUT1 mRNA expression among DCM, IHD and IHD-T2DM patients (Fig. 5b). Similarly, GLUT4 expression was not significantly affected by CRT in any of the above groups (Fig. 5c). These remained true even after adjusting for age, sex, and BMI.

## Discussion

This is the first study to provide a comprehensive evaluation of myocardial LV SGLT1 expression in context of the other two major myocardial glucose transporters, GLUT1 and GLUT4, in a relatively large number of myocardial LV human samples representing patients with end-stage HF. We identified that (i) LV SGLT1 expression shows differences according to HF etiology, (ii) SGLT1 expression positively correlates with LV dilation (LVEDD) and negatively correlates with LV systolic function (EF), and (iii) CRT affects SGLT1 expression mainly in patients with DCM.

In large cardiovascular outcome trials, SGLT2 inhibitors consistently reduced the risk of hospitalization for HF in T2DM patients [1–3]. Those with HF at baseline drived the most benefit [37], fueling speculations regarding the mechanism of cardioprotection mediated by SGLT2 inhibitors. However, in the DAPA-HF and the EMPEROR-Reduced trials—two dedicated HF trials—the SGLT2 inhibitors dapagliflozin and empagliflozin reduced hospitalization for HF similarly in non-diabetic and diabetic HFrEF patients [5, 6]. These data suggest that SGLT2 inhibitors have direct cardioprotective effects in HF, which are independent of the presence of T2DM [38, 39]. The mechanism of such direct cardioprotection exerted by SGLT2 inhibitors is unclear, since SGLT2 is not expressed in the heart [15, 16]. Accordingly, in the present study we could not detect myocardial LV SGLT2 mRNA expression in controls or in those with end-stage HF. On the contrary, SGLT1 has been recently identified as a highly expressed, major glucose transporter in the heart alongside GLUT1 and GLUT4 [17–21]. In cases when upregulated, dual SGLT1/2 inhibitors and even SGLT2 inhibitors might modulate SGLT1 in the heart, but this is currently unclear [8]. Yet, not only the relevance of SGLT1 inhibition in the heart has been ill-defined, but also the characterization of myocardial LV SGLT1 expression on representative and large number of samples from human patients with end-stage HF has been limited so far.

In the present study, we document that SGLT1 expression shows characteristic changes according to HF etiology. In HCM patients, LV SGLT1 expression was similar to that of controls. A previous study found that HCM was associated with a modest ~ 1.4-fold upregulation of SGLT1 compared with controls [16]. Notably, LV septal samples from those HCM patients were harvested during surgical septal myectomy [16]. While the functional characteristics of those patients were not assessed, it is possible that they were not in an end-stage HF phase at the time of sample collection, unlike our HCM patients, who had severely reduced EF compared to controls. On the other hand, our results show that LV SGLT1 expression was significantly upregulated in patients with DCM. This seemingly contradicts the findings of Banerjee et al. [18] who documented no significant upregulation of SGLT1 in DCM patients. However, it is unclear from their study how many of those DCM patients were on CRT prior to LV sample collection. We show that CRT was associated with significant reduction in LV SGLT1 expression in patients with DCM. Nonetheless, Lambert et al. [24] also identified a significant upregulation of SGLT1 protein expression in a group of HF patients, the majority of whom had DCM. Likewise, we found that LV SGLT1 expression was upregulated in patients with IHD, in good agreement with previous reports in humans with HF [16, 18].

In the murine heart, acute myocardial ischemia was shown to upregulate SGLT1 [18, 40], whereas permanent left anterior descending coronary artery ligation resulted in increased SGLT1 expression in the intact LV area [41], suggesting that SGLT1 might have a causal role in the development of IHD. However, pharmacological modulation of SGLT1 in acute ischemia has been controversial. The non-selective SGLT1/2 inhibitor phlorizin exacerbated LV dysfunction following ex vivo myocardial ischemia–reperfusion injury in mice via inhibiting glucose uptake, suggesting that SGLT1 might play a compensatory protective role in acute ischemia [19, 21]. In murine models of IHD, one study found that dual SGLT1/2 inhibition impaired myocardial function [42], whereas others reported protective effects [41, 43]. While possible differences related to off-target mechanisms and dosages of the medications cannot be ruled out, cardiomyocyte-specific knock-down of SGLT1 in mice reduced infarct size following myocardial ischemia–reperfusion injury in vivo and ex vivo [40]. The knock out of SGLT1 also protects against ischemia–reperfusion injury in the kidney [44].

In the present study, we observed that non-diabetic and diabetic IHD patients showed comparable upregulation of LV SGLT1 expressions, indicating that T2DM itself might not further increase the already elevated SGLT1 expression in HF. Indeed, in a previous study, LV SGLT1 expression was elevated to similar extent in patients with diabetic cardiomyopathy and IHD, respectively, compared with controls [24]. While the role of SGLT1 in ischemic injury remains controversial, growing body of evidence suggest that SGLT1 might promote cellular injury in hyperglycemic states. Insulin-induced cardiac glucose uptake was shown to be mediated predominantly by SGLT1 in healthy mice [18] and in human failing cardiomyocytes [17]. In cardiomyocytes from rats with T2DM, this increase in SGLT1-dependent glucose uptake was coupled with elevation of intracellular sodium ion content [24]. The latter is a shared pathology in HF patients with and without diabetes [8]. In response to high glucose concentrations, SGLT1 contributed to cellular glucotoxicity in cultured rat cardiomyocytes via increased activation of NAPDH-oxidase [45], whereas it facilitated profibrotic signaling in human cardiac fibroblasts [46]. Nonetheless, in the present study, immunohistochemical staining of SGLT1 suggested that cardiomyocytes—but not fibrotic tissue—are the primary sources of SGLT1 expression in the myocardium, in line with Kashiwagi et al. [19]. Furthermore, the presence of SGLT1 almost exclusively corresponded to that of Na–K-ATPase in cardiomyocytes, indicating that it is localized in the sarcolemma, as previously suggested in mice [18, 41] and in human hearts [16].

This is the first study to document a significant positive correlation between LV SGLT1 expression and LVEDD in humans with HF, hinting that SGLT1 expression might be related to LV dilation. Furthermore, SGLT1 expression negatively correlated with systolic function characterized by EF and FS. While a causal relationship between SGLT1 expression and LV adverse remodeling cannot be inferred based on our present study, mounting evidence suggest that—unlike in acute phases—chronic upregulation of SGLT1 might promote maladaptive changes. Cardiomyocyte-specific overexpression of SGLT1 in non-diabetic mice resulted in development of cardiac hypertrophy and failure, characterized by accumulation of fibrosis, increased LVEDD and reduced FS proportionately with the increase in the expression of SGLT1 [20]. Interestingly, when suppressing SGLT1 expression after 10 weeks of overexpression, cardiac structure (LVEDD) and function (FS) returned to normal [20]. Hence, upregulation of SGLT1 alone might be sufficient to induce HF, while blockade of SGLT1 rescues the HF phenotype by eliciting reverse remodeling.

The regulation of SGLT1 expression in the heart remains to be further elucidated. In mice, overactivation of myocardial AMPK promoted glycogen accumulation and LV remodeling through increased SGLT1 activity [20]. In the present study, LV AMPKα phosphorylation increased in HF patients with DCM, and IHD, which reached statistical significance in IHD-T2DM patients. Its expression negatively correlated with LV systolic function and was significantly associated with upregulation of LV SGLT1 expression. These suggest that AMPK might be implicated in upregulation of SGLT1 protein expression, in line with small animal studies [20, 40, 47]. In contrast, ERK1/2 phosphorylation was significantly downregulated in patients with DCM, IHD and IHD-T2DM compared to non-failing controls, whereas deterioration in LV systolic function (EF) was associated with the reduction in the activating phosphorylation of ERK1/2. This is in agreement with studies in mice and humans, demonstrating that end-stage HF was coupled with substantial decrease in ERK1/2 phosphorylation compared to healthy littermates and non-failing controls, respectively [48, 49]. In our study, this reduced ERK1/2 phosphorylation in patients with HF was accompanied by the counter-upregulation of SGLT1 expression, showing significant negative correlation. Interestingly, SGLT1 knock down in mice itself was sufficient to upregulate ERK1/2 phosphorylation and to prevent the development of HF in response to aortic constriction [50]. Vice versa, reduction in SGLT1 expression in primary cultured rabbit renal proximal tubule cells was dependent on increased ERK1/2 activity [51–53]. These, together with our findings might imply that ERK1/2 is a negative regulator of SGLT1.

In relation to these, we documented that CRT was associated with reduced SGLT1 expression in HF patients compared to those not on CRT, specifically in those with DCM. It is well documented that CRT exerts beneficial molecular, LV structural and functional effects especially in patients with DCM [34]. We should point out that all HF patients in our study had end-stage disease, even those with CRT. However, restoration of ventricular synchrony with CRT could have beneficial molecular effects beyond anatomical and functional improvements, which needs to be further elucidated. Despite similar LV dilation and EF values, DCM patients with CRT had significantly lower LV SGLT1 mRNA expression compared to those not on CRT, independent of age, sex, and BMI. Conversely, ERK1/2 phosphorylation was found to be significantly upregulated, which further reinforces that ERK1/2 could be a negative regulator of SGLT1 in the heart.

We assessed LV mRNA expression of the other two major glucose transporters. We found that despite a ~ twofold upregulation of SGLT1, the mRNA expression of GLUT1 was only slightly increased compared with controls (after adjusting for baseline characteristics) and we found no correlation between SGLT1 and GLUT1 expressions in end-stage HF patients. In mice with cardiomyocyte-specific SGLT1 knock down, the expression of GLUT1 did not show any compensatory alterations [20]. These might suggest that SGLT1 and GLUT1 play a different role in HF. Indeed, in contrast to SGLT1, cardiomyocyte-specific GLUT1 overexpression prevented the development of HF following aortic constriction in mice [54]. Hence, chronic upregulation of GLUT1—unlike that of SGLT1—could be a compensatory mechanism in HF. Importantly, in mouse models, manipulation of SGLT1 and GLUT1 expressions were accompanied by unaltered GLUT4 expression. In line with a previous study in humans [17], we also found that GLUT4 mRNA expression was unaltered in end-stage HF patients compared with controls, however, we did not quantify its expression separately in the membrane fraction which remains a limitation. Finally, CRT had no effect on GLUT1 and GLUT4 mRNA expressions in any of the HF etiological subtypes.

## Conclusions

In conclusion, LV SGLT1 expression shows etiology-dependent alterations in human patients with end-stage HF, and correlates significantly with LV dilation and with the deterioration of LV systolic function. AMPK and ERK1/2 might play antagonistic role in the regulation of SGLT1 expression. The potential causal role of SGLT1 in HF development and whether its pharmacological blockade exerts cardioprotection need to be further elucidated.

## Data Availability

The datasets used and/or analyzed during the current study are available from the corresponding author on reasonable request.

## Abbreviations

AMPKα: Adenosine-monophosphate-activated protein kinase catalytic α subunit
ANCOVA: Analysis of covariance
ANOVA: Analysis of variance
BCa 95% CI: Bias-corrected and accelerated 95% confidence intervals
CRT: Cardiac resynchronization therapy
DAPA-HF: Dapagliflozin and Prevention of Adverse Outcomes in Heart Failure
DAPI: 4′,6-Diamidino-2-phenylindole
DCM: Dilated cardiomyopathy
EF: Ejection fraction
EMPEROR-Reduced: Empagliflozin outcome trial in patients with chronic heart failure with reduced ejection fraction
ERK1/2: Extracellular signal-regulated kinase 1/2 (p44/42 mitogen-activated protein kinase, MAPK)
GAPDH: Glyceraldehyde-3-phosphate dehydrogenase
GLUT1 or GLUT4: Facilitative glucose transporter 1 or 4
HCM: Hypertrophic cardiomyopathy
HF: Heart failure
HFrEF: Heart failure with reduced ejection fraction
HTx: Heart transplantation
IHD: Ischemic heart disease
LV: Left ventricle or left ventricular
LVAD: Left ventricular assist device therapy
LVEDD: Left ventricular end-diastolic diameter
LVESD: Left ventricular end-systolic diameter
Na–K-ATPase: Sodium–potassium ATPase
qRT-PCR: Quantitative real-time polymerase chain reaction
RIN: RNA integrity number
SGLT1 or SGLT2: Sodium-glucose cotransporter 1 or 2
SLC2A1 or SLC2A4: Solute Carrier Family 2 Member 1 or 4
SLC5A1 or SLC5A2: Solute Carrier Family 5 Member 1 or 2
T2DM: Type 2 diabetes mellitus
